# Cystometric Measurements in Rats with an Experimentally Induced Traumatic Brain Injury and Voiding Dysfunction: A Time-Course Study

**DOI:** 10.3390/brainsci9110325

**Published:** 2019-11-14

**Authors:** Chellappan Praveen Rajneesh, Ling-Yu Yang, Shih-Ching Chen, Tsung-Hsun Hsieh, Hung-Yen Chin, Chih-Wei Peng

**Affiliations:** 1 School of Biomedical Engineering, College of Biomedical Engineering, Taipei Medical University, Taipei-11031, Taiwan; 2 Department of Physical Medicine and Rehabilitation, School of Medicine, College of Medicine, Taipei Medical University, Taipei-11031, Taiwan; 3 Department of Physical Medicine and Rehabilitation, Taipei Medical University Hospital, Taipei-11031, Taiwan; 4 Department of Physical Therapy and Graduate Institute of Rehabilitation Science, College of Medicine, Chang Gung University, Taoyuan-33302, Taiwan; 5 Neuroscience Research Center, Chang Gung Memorial Hospital, Linkou-33305, Taiwan; 6 Department of Obstetrics and Gynecology, Taipei Medical University Hospital, Taipei-11031, Taiwan; 7 Department of Obstetrics and Gynecology, School of Medicine, College of Medicine, Taipei Medical University, Taipei-11031, Taiwan; 8 Research Center of Biomedical Device, Taipei Medical University, Taipei-11031, Taiwan

**Keywords:** traumatic brain injury, cystometric measurements, time-course analysis, external urethral sphincter electromyographic activity

## Abstract

Traumatic brain injuries (TBIs) are a serious public health issue worldwide with increased mortality as well as severe disabilities and injuries caused by falls and road accidents. Unfortunately, there is no approved therapy for TBIs, and bladder dysfunction is a striking symptom. Accordingly, we attempted to analyze bladder dysfunction and voiding efficiency in rats with a TBI at different time-course intervals. Time-dependent analyses were scheduled from the next day until four weeks after a TBI. Experimental animals were grouped and analyzed under the above conditions. Cystometric measurements were used for this analysis and were further elaborated as external urethral sphincter electromyographic (EUS-EMG) activity and cystometrogram (CMG) measurements. Moreover, magnetic resonance imaging (MRI) studies were conducted to investigate secondary injury progression in TBI rats, and results were compared to normal control (NC) rats. Results of EUS-EMG revealed that the burst period, active period, and silent period in TBI rats were drastically reduced compared to NC rats, but they increased later and reached a stagnant phase. Likewise, in CMG measurements, bladder function, the voided volume, and voiding efficiency decreased immediately after the TBI, and other parameters like the volume threshold, inter-contraction interval, and residual volume drastically increased. Later, those levels changed, and all observed results were compared to NC rats. MRI results revealed the prevalence of cerebral edema and the progression of secondary injury. All of the above-stated results of the experiments were extensively substantiated. Thus, these innovative findings of our study model will surely pave the way for new therapeutic interventions for TBI treatment and prominently highlight their applications in the field of neuroscience in the future.

## 1. Introduction

Traumatic brain injuries (TBIs) are a foremost cause of disability and mortality worldwide. TBI is defined as an alteration in brain function, or other evidence of brain pathology, caused by an external force [1]. Over 2 million people around the world are severely affected by a TBI annually. Apart from financial burdens and mortality, the effects of TBI also extend to permanent impairment of behavioral, cognitive, and emotional activities [2]. In both developed and underdeveloped countries, motor vehicles are the major cause of death and disabilities, particularly in young people [3]. TBI is a multifactorial event with a cascade of complex pathophysiological processes. TBI not only causes structural damage but also induces functional deficits that are due to both primary and secondary injury mechanisms. The neurological damage does not occur immediately after the moment of impact (primary injury) but gradually develops afterwards (secondary injury). It is evident that secondary brain injury is the leading cause of hospital deaths according to the nature and severity of the impact [4]. Secondary injury may evolve over minutes to months after the primary injury and is the result of a cascade of metabolic, cellular, and molecular events that ultimately lead to brain cell death, tissue damage, and atrophy [5].

Secondary brain injury is a critical phase and is mainly caused by brain swelling, which increases the pressure inside the skull and subsequently decreases the blood flow to the brain, leading to an ischemic condition [6]. Cerebral herniation is a process that mainly is due to the condition of ischemia or an increase in the intracranial pressure. It is well documented that cerebral herniation increases brain damage and morbidity [3]. In the acute stage of TBI, the common cognitive outcomes are decreased speed of information processing, attention problems, and confusion and in the chronic stage the outcomes extend to dizziness, sleep disturbance, anxiety, and depression. This chronic condition leads to the risky state of post-concussive syndrome [7,8]. Numerous methods are used to analyze outcomes of a TBI such as the fluid percussion model [9], weight-drop model (WDM) [10], controlled cortical-impact (CCI) model [11], and so on. Newer research in this field mainly focuses on associating behavioral patterns of patients with physical injuries, analyzing tissue recovery, investigating how to compensate for memory loss, and also growing stem cells in lesional regions [2]. Studies revealed that TBIs that occur in the area of the pontine micturition center (PMC) interrupt the supraspinal regulation of bladder function [12]. Henceforth, fundamental bladder problems are characterized early after a TBI. Since motorcycle accidents are a major cause of TBIs, the present study mimics the same method. Rodents have predominantly been used in urodynamic studies to analyze urinary storage and voiding functions, in both normal animals and experimental subjects [13,14]. In the present study, the WDM was used to establish TBIs. After experiencing a TBI, animals may develop abnormal bladder function, and this was analyzed by cystometric measurements at different time periods.

## 2. Methodology

### 2.1. Experimental Animals

Forty-two male Sprague Dawley rats (experimental subject *n* = 36; normal control (NC) *n* = 6) (BioLASCO Taiwan, Yilan, Taiwan) weighing 270–320 g were utilized for the present experiment. All experimental protocols used in the current study were approved by the Institutional Animal Care and Use Committee (IACUC) of Taipei Medical University (TMU) and followed TMU IACUC guidelines for treating animals humanely and reducing animal suffering by using appropriate anesthesia and analgesics (IACUC approval no. LAC-2013-0199). The animal house was well-equipped with temperature- and humidity-controlled instruments, and all animals were kept in the animal house and were well maintained according to ethical standards. All animals were placed under a 12 h light/dark cycle with food and water available ad libitum. In addition, at the end of the experiment, all animals were sacrificed by carbon dioxide inhalation followed by cervical dislocation.

### 2.2. Induction of TBI

All experiments were performed in the daytime. Animals were anesthetized for less than 10 min by using 3% isofurane (Aesica, Queenbrough, UK) 1–2 min prior to impact in order to reduce the suffering and distress during the induction of the TBI. Marmarou’s impact acceleration model was employed to induce a TBI with minimal modifications [10,15] by freely dropping a 450 g brass weight from a 2 m height through a vertical transparent Plexiglas tube. After the impact, ventilator support (Harvard Rodent Ventilator, model 683, South Natick, MA) with a mixture of 30% O_2_ + 70% N_2_O was immediately initiated with the purpose of reducing the mortality rate. Both NC and experimental rats underwent the same type of surgical procedures, except that NC rats did not receive the WDM model of TBI induction. The body temperature of rats was maintained at 37.0 ± 0.5 °C using an adjustable heating pad during recovery from anesthesia, and the body temperature was constantly monitored throughout surgery using a rectal probe. Except for the NC group, all animals were separated into four groups consisting of nine animals (*n* = 9) each. The time-course study was mainly conducted to evaluate changes in bladder function and voiding efficiency (VE) as secondary injury developed in TBI rats. Hence, each group underwent experiments on different schedules. Group 1 was analyzed within 1 d after the TBI, and similarly group 2 was analyzed at the end of week 1, group 3 at week 2, and group 4 at week 4 after TBI induction.

### 2.3. Impact Assessment of TBI Rats by Time-Course Cystometric Measurements

To investigate the TBI effects in the experimental and control animals, a cystometric analysis was performed. According to the different groups, the experimental procedures were scheduled as described above. The animals were anaesthetized by using urethane (1.25 g/kg, subcutaneous). An animal was placed on a heated pad, and the urinary bladder and distal part of the urethra were exposed through an abdominal incision. A polyethylene (PE) tube 60 (1.0 mm inside diameter and 1.5 mm outside diameter) was inserted into the bladder lumen for bladder pressure measurements. The bladder end of the PE tube was heated to form a collar and then passed through a small incision at the apex of the bladder dome, and the tube was secured with a purse-string suture. For recording external urethral sphincter electromyographic (EUS-EMG) activity, a couple of 50 µm epoxy-coated platinum–iridium wire electrodes (A-M systems, Everett, WA, USA) were hooked at the tip of a 30-gauge needle and inserted in to the both sides of EUS under direct visual examination. The needles were withdrawn, and the electrodes were left embedded in the muscle. The abdominal wall was closed with nylon sutures allowing the wire electrodes and bladder catheter to exit. The PE tube was, in turn, connected via a 3-way stopcock to an infusion pump for filling with physiological saline and to a pressure transducer for monitoring the intravesical pressure (IVP). Indeed, the wire electrodes were connected to a computer where the cystometrogram (CMG) and EUS-EMG data were amplified and digitized. The analyst who assessed the bladder activity was blinded to the status of the rat. To observe bladder activity, saline was instilled into the bladder by the PE 60 tube controlled by a saline injector at a rate of 0.12 mL/min. Meanwhile, bladder activity was monitored by CMG readings, and simultaneous EUS-EMG activity was static. While voiding, EUS-EMG activity was observed as a result of the burst activity of the EUS.

In the course of voiding, the bladder begins to contract, and the external urethral sphincter begins to discharge large amounts of urine. During discharge, if the timescale is further enlarged, one can observe that there are many active periods (APs) inside the burst period (BP), and high-frequency discharges will be separated by a silent period (SP). For the time-course analysis, several EUS-EMG parameters were measured. The BP can be defined as the beginning of conversion of tonic EMG into the burst discharge, which ends when the EMG is converted to tonic EMG, and it includes both the SP and AP. The SP was defined as the inert period that exists between two high-frequency spikes, which can be further well-defined as a static period between the conversion point of high-frequency spikes into low-frequency waves and vice versa. APs are high-frequency spikes that are separated by an inert period, and an AP can also be clearly defined as the period between which low-frequency waves are converted to high-frequency spikes and vice versa.

In addition, several CMG parameters were measured to quantify the effects of the TBI on voiding function such as the micturition volume threshold (VT), defined as the infused volume of saline sufficient to induce the first voiding contraction; the contraction amplitude (CA), defined as the maximum pressure during voiding; and the bladder contraction duration (CD) during voiding. The VE is the ratio of the voided volume (VV) to the VT. The VV was calculated as the VT minus the residual volume (RV) of saline withdrawn through the intravesical catheter after the final voiding contraction. Both EUS-EMG and CMG parameters were calculated using Acknowledge software (Biopac Systems, Inc., Goleta, CA, USA).

### 2.4. Magnetic Resonance Imaging (MRI) to Assess Secondary Injury Progression in Time-Course Intervals

In order to assess time-course morphological alterations in the brain, MRI was performed. The MRI study was carried out on NC and experimental TBI rats. For TBI rats, MRI was performed at particular time intervals from the day after the impact (day 1), and at weeks 1, 2, and 4. Three live animals from each group (*n* = 3) were subjected to this study from different time-course interval groups (day 1, and weeks 1, 2, and 4). Rats were anesthetized with tiletamine zolazepam (50 mg/kg, i.p.), and sedated rats were placed in a prone position in a 7T Bruker PharmaScan 70/160US (Bruker Medical System, Karlsruhe, Germany). All MRI data were processed using Paravision 6.0 software (Bruker Medical System). The same abovementioned procedure was performed on NC rats (*n* = 3) without TBI induction.

### 2.5. Statistical Analysis

Results are presented as the mean ± standard deviation (SD), and data were analyzed by a one-way analysis of variance (ANOVA) for overall comparisons between NC and TBI rats (time-course analysis) followed by Tukey’s post hoc test. *p* values of <0.05 were considered statistically significant. Statistical analysis was performed using GraphPad Prism 6 software (GraphPad Software, San Diego, CA, USA).

## 3. Results

### 3.1. Detailed Time-Course Study of EUS-EMG Activity in Experimental TBI Rats

A TBI was induced by the WDM from a 2 m height. Typically, experimental rats showed variable periods of convulsions and apnea following the impact. The experimental rats which did not spontaneously recover from the trauma were not resuscitated and were excluded from the analysis. In the entire study, only two animals belonging to the day 1 group died immediately after the impact, even before conducting other experiments. Remaining animals survived to the end of the experiments. The animals received no ventilator support at any time during the procedure, and surviving rats were further subjected to cystometric measurements at different time intervals, including on day 1, which was considered to be the immediate day following the TBI, and at weeks 1, 2, and 4.

**Figure 1:**
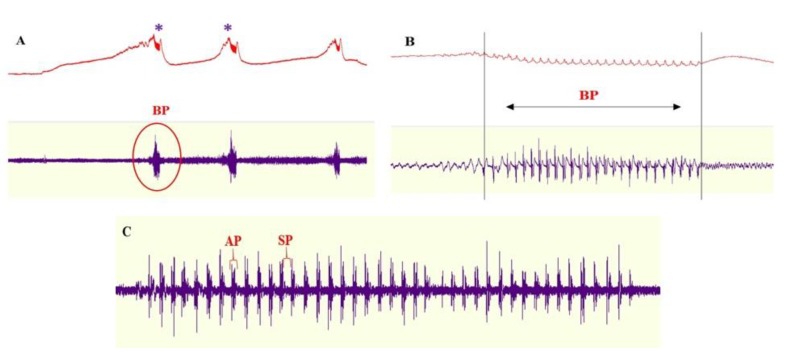
(**A**) Typical cystomyogram pattern indicating the burst period (BP) of normal control (NC) rats. (**B**) The burst period (BP) in the external urethral sphincter electromyography (EUS-EMG). (**C**) Individual bursts consisting of active (AP) and silent periods (SP).

**Scheme 1:**
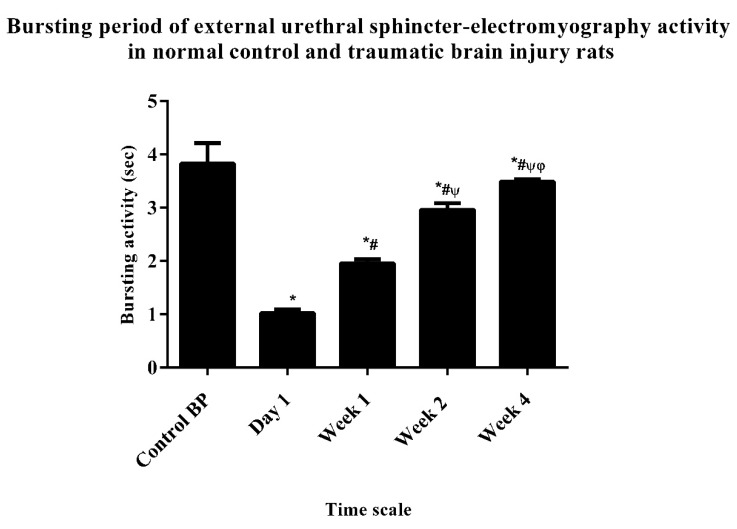
Illustration of the time-course comparisons of the burst period between the normal control (NC) rats and rats with an experimentally induced traumatic brain injury (TBI). An asterisk (*) indicates a significant difference compared to the NC group. “#” indicates a significant difference of experimental groups compared to the TBI day-1 group of animals. “^ψ^” indicates a significant difference of experimental groups compared to the TBI week-1 group of animals. “^φ^” indicates a significant difference of experimental groups compared to the TBI week-2 group of animals. Significance levels are marked at *p* < 0.05.

Cystometric measurements were elaborated as EUS-EMG and CMG. The illustrations furnished in Figure 1 show the typical pattern of cystomyogram of NC rats, and it also depicts the patterns of EUS-EMG, BP, AP, and SP. The EUS-EMG results furnished in Scheme 1 illustrate a comparison of the BPs between NC (*n* = 6) and experimental TBI rats (*n* = 34). Interestingly, the BP of all TBI groups of animals exhibited a significant difference against NC rats, except for the week-4 group of TBI animals. The observed EUS-EMG results revealed that, eventually, the BP of experimental TBI animals rapidly decreased in the day-1 and week-1 groups, and the day-1 group of animals displayed significant differences with the week-1, -2, and -4 groups of animals. Likewise, the week-1 group of animals significantly differed from the week-2 and -4 groups of animals. In addition, the week-2 group of animals exhibited an elevated BP level compared to the day-1 and week-1 groups of animals, and it significantly differed only from the week-4 group of TBI animals. Finally, the week-4 group of animals significantly differed from all other TBI groups of animals. Interestingly, the week-4 group of animals also showed an elevated BP level compared to all other TBI groups of animals.

**Scheme 2:**
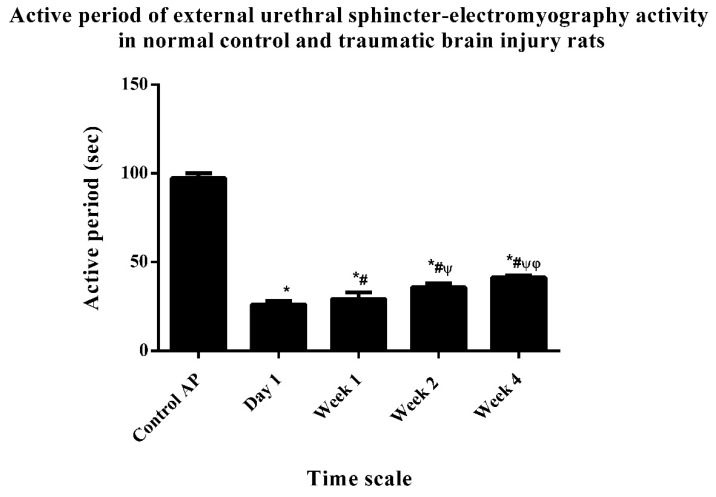
Illustration of time-course comparisons of the active period between the normal control (NC) rats and rats with an experimentally induced traumatic brain injury (TBI). An asterisk (*) indicates a significant difference compared to the NC group. “#” indicates a significant difference of experimental groups compared to the TBI day-1 group of animals. “^ψ^” indicates a significant difference of experimental groups compared to the TBI week-1 group of animals. “^φ^” indicates a significant difference of experimental groups compared to the TBI week-2 group of animals. Significance levels are marked at *p* < 0.05.

Scheme 2 clearly depicts that AP levels also showed a constant decrease in the day-1 and week-1 groups of animals. There was a significant difference (*p* < 0.05) observed in both the day-1 and week-1 groups of animals compared to NC animals. Apart from this, week-2 and -4 groups of animals gradually showed an elevated pattern of values compared to the other TBI groups of animals, and they also exhibited a significant difference from the NC group. The day-1 group of animals showed significant differences from week-1, -2, and -4 groups of experimental TBI animals. The week-1 group of animals exhibited significant differences from the week-2 and -4 groups of animals. The week-2 group of animals demonstrated a significant difference from the week-4 group of animals. Finally, the group comparison results revealed that the week-4 group of animals significantly differed from all other TBI groups of animals.

**Scheme 3:**
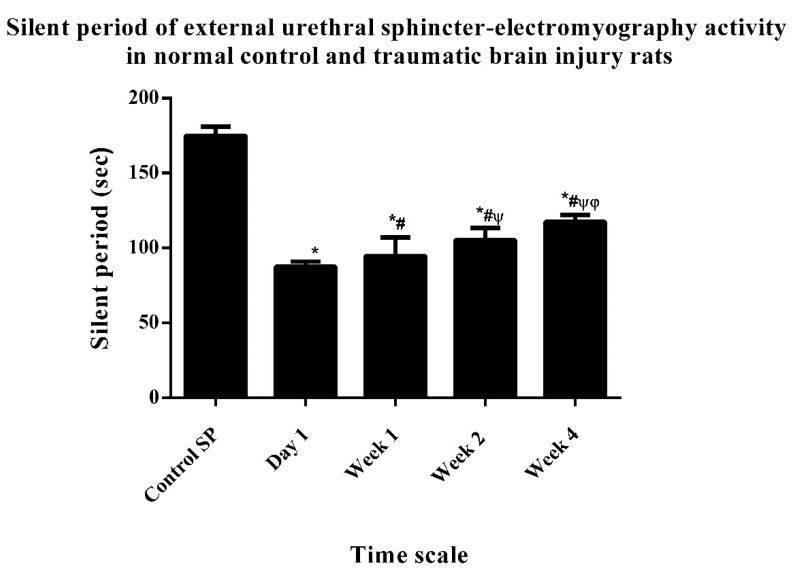
Illustration of the time-course comparison of the silent period between normal control (NC) rats and rats with an experimentally induced traumatic brain injury (TBI). An asterisk (*) indicates a significant difference compared to the NC group. “#” indicates a significant difference of experimental groups compared to the TBI day-1 group of animals. “^ψ^” indicates a significant difference of experimental groups compared to the TBI week-1 group of animals. “^φ^” indicates a significant difference of experimental groups compared to the TBI week-2 group of animals. Significance levels are marked at *p* < 0.05.

Scheme 3 portrays values of the SP in EUS-EMG activity of NC and TBI rats. SP levels also exhibited a sudden drop immediately after the impact in day-1 animals and further exhibited a gradual elevation in other groups such as in weeks 1, 2, and 4 with maximum elevated levels. No elevated levels were above the NC level of the SP; indeed, all SP levels of TBI animals showed a significant difference with the NC group. The day-1 group of animals exhibited a substantial difference from all other TBI group of animals. The week-1 group of TBI animals showed important differences with week-2 and -4 groups of TBI animals. The week-2 group of animals exhibited a significant difference from the TBI week-4 group of animals. Similarly, like the above-mentioned AP values, group comparison results of SP also revealed that only the week-4 group of animals significantly differed from all of the other TBI groups of animals. Neither the SP nor AP levels of the week-4 group of TBI animals exhibited a prominent elevated value compared to the week-3 group of animals.

### 3.2. Comprehensive Time-Course Study of CMG Measurements in Experimental TBI Rats

Results furnished in Table 1 summarize CMG observations of TBI animals compared to NC rats and among the experimental groups. The VT drastically increased in all TBI animals in all time-course intervals, except the week-2 group of animals (0.66 ± 0.06 mL), which showed a decrease compared to other TBI animals. All of the other TBI groups of animals revealed a significant difference compared to NC rats, and the day-1 group of TBI animals exhibited a significant difference from the week-4 group of animals. In addition, a noteworthy variance was observed in the week-2 and -4 groups of animals, and VT values we observed were highly and significantly different from day-1 and week-1 groups of animals. Results of CA displayed a considerable decrease in TBI animals at all time-course intervals, excluding the week-4 group of animals. The week-4 group of TBI animals possessed a mildly elevated value of CA (28.00 ± 5.55 cm H_2_O) compared to CA values of other TBI animals, and all groups of animals exhibited significant differences against the NC group (35.21 ± 2.36 cm H_2_O). CA results of the day-1 group of animals (26.17 ± 4.10 cm H_2_O) substantially differed from results of the week-1 group of animals but not with the week-2 or -4 groups of TBI animals. Likewise, CA values of the week-1 group of animals (24.79 ± 5.33 cm H_2_O) significantly differed from CA values of the week-2 and -4 groups of TBI animals, but not with the day-1 or week-1 groups of animals. Fascinatingly, CA values of the week-2 group of animals (27.67 ± 3.56 cm H_2_O) did not significantly differ from the week-4 group of animals (28.00 ± 5.55 cm H_2_O) or with other groups of TBI animals.

The CD invariably and significantly decreased in all TBI groups compared to the NC group at 20.64 ± 2.82 s. In the day-1 group, the level was 16.23 ± 3.22 s, and it only significantly varied with the week-1 group of TBI animals. The week-1 group of animals had a level of 15.93 ± 2.62 s, and it significantly differed from the week-2 group of TBI animals. Compared to all TBI groups, the week-2 group of TBI animals illustrated a decreased level of CD (14.85 ± 3.06 s), and it only significantly differed from the week-4 group of TBI animals. Nevertheless, the week-4 group animals showed a value of 15.78 ± 3.02 s for CD, which exhibited significant differences with the NC group and week-2 TBI animals, but not with the day-1 or week-1 groups of TBI animals.

The inter-contraction interval (ICI) of the NC group was 68 ± 17 s; after the TBI, all groups of experimental animals showed incredibly elevated and significant values of ICI compared to the NC group. Animals immediately after the day of impact presented a value of 108 ± 20 s, and this did not significantly differ from other TBI groups of animals, except with the week-4 group of TBI animals. The week-1 group of animals (108 ± 18 s) had an almost similar value with the day-1 group of animals, and the value of the week-1 TBI group of animals did not significantly differ from day-1 or week-2 groups of TBI animals, but it highly and significantly differed from the week-4 group of TBI animals. Moreover, ICI values began to drop in the day-1 and week-1 groups of animals compared to week-2 (101 ± 36 s) and -4 (96 ± 25 s) TBI groups of animals. The week-2 group of TBI animals exhibited no significant difference with other groups of TBI animals, but the value of the week-4 group of animals was lower than values of the NC group and day-1 and week-1 groups of TBI animals.

The RV of the NC group was 0.18 ± 0.04 mL, and values obtained from TBI animals with different time-course intervals were significantly higher. The day-1 and week-1 groups of animals had values of 0.59 ± 0.07 and 0.51 ± 0.05 mL, respectively. In this context, the day-1 group of animals showed a significant difference compared to all other groups of TBI animals. Likewise, the week-1 group of animals also revealed a significant difference with other groups of TBI animals except for the day-1 group of TBI animals. TBI animals from the week-2 and -4 groups showed mildly lower values of 0.46 ± 0.09 and 0.28 ± 0.03 mL, respectively, compared to other TBI animals, and the week-2 group of animals showed a significant difference against all other groups of animals except the day-1 group of TBI animals. Nevertheless, values of day-1 and week-1 and -2 groups of animals significantly differed from the week-4 group of TBI animals.

The observed VV in the NC group was 0.25 ± 0.02 mL, and initially, the VV of TBI animals precipitously dropped. All values obtained from TBI animals showed significant differences with NC animals. Day-1 TBI animals showed a very low volume of 0.10 ± 0.02 mL, and values significantly differed from all other groups of TBI animals. Week-1 TBI animals exhibited a volume of 0.12 ± 0.02 mL, and this value significantly differed from all other TBI groups of animals except for the day-1 group of animals. In addition, TBI animals of weeks 2 and 4 showed higher values of RV at 0.20 ± 0.05 and 0.22 ± 0.04 mL, respectively, and the value of the week-2 group significantly differed from the week-4 group of animals. Week-4 TBI animals exhibited higher values of VV compared to other TBI animals. However, day-1 and week-1 and -2 groups of animals significantly differed from the week-4 group of TBI animals.

Finally, the VE of NC animals was 66.4% ± 7%, and results of all other TBI animals were drastically lower compared to the NC group. Nonetheless, all TBI animals displayed a significant difference with the NC group of animals. The day-1 group of animals revealed a very low VE of 14.4% ± 5%, and that value significantly differed from other TBI groups of animals. The week-1 group of animals also presented an almost similar value of 16.8% ± 5%, and it significantly differed from values of the week-2 and -4 groups of TBI animals. TBI animals from the week-2 group exhibited a fair improvement in VE of 31.2% ± 8%, and this value of the week-2 group of animals did not substantially differ from other groups of TBI animals. TBI animals in the week-4 group showed a maximum and stable VE of 32.6% ± 6%, and all the other TBI groups and NC only significantly differed from the week-4 TBI group but not the week-2 group of TBI animals. Animals belonging to all groups (day 1, week 1, and week 2) exhibited a significantly different VE compared to the NC group, especially the week-4 group of animals, which confirmed a stable condition with remarkable VE compared to the other TBI animals. This was further substantiated by results that the difference in VE between the week-2 and -4 groups of animals was very small; thus, there was little chance for improvement in the VE.

### 3.3. MRI Studies

**Figure 2:**
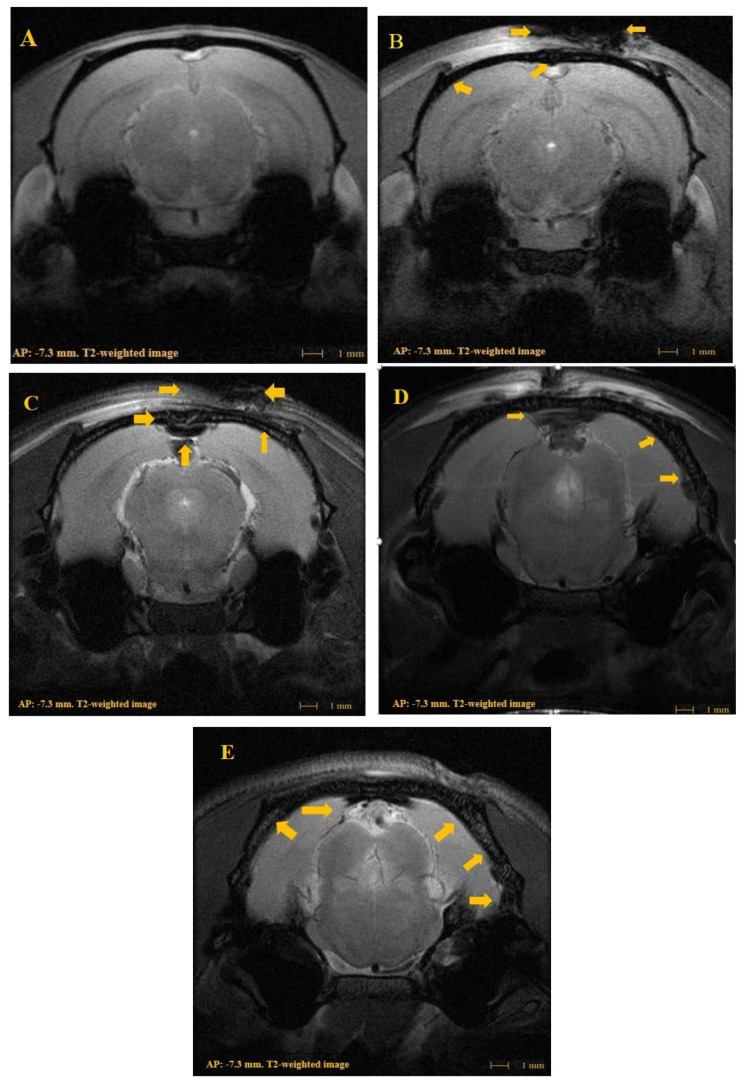
(**A**) T2-weighted magnetic resonance imaging (MRI) of normal control (NC); (**B**) T2-weighted MRI of a rat with an experimentally induced traumatic brain injury (TBI) on day 1; (**C**) T2-weighted MRI of a rat with a TBI at week 1; (**D**) T2-weighted MRI of a rat with a TBI at week 2; (**E**) T2-weighted MRI of a rat with a TBI at week 4. Arrows indicate the severity of the TBI and cerebral edema; all TBIs shown here were induced by a weight-drop model at a 2 m height.

MRI observations clearly illustrated time-course morphological alterations, impacts of the TBI, and the progressive development of secondary injury in the brain. The time-course study of the brain and respective MRI images clearly revealed the prevalence of cerebral edema and intracranial hemorrhaging close to the cerebral cortex due to the weight drop inducing the TBI. The MRI image in Figure 2A demonstrates the normal condition of the rat brain, and in the corresponding MRI images of 2B (day1), 2C (week 1), 2D (week 2), and 2E (week 4), the arrow indicates cerebral edema and the progression of intracranial hemorrhaging of TBI rats in time-course intervals.

## 4. Discussion

There is much scientific literature available related to the WDM for inducing TBI, and both WDM and TBI have also been extensively studied. Hitherto, there were very few TBI studies related to bladder function and urinary incontinence, while those pertaining to a time-course analysis and bladder dysfunction are also very rare. Hence, this study attempted to evaluate the urodynamic status of TBI rats as secondary injury progressed by means of time-course studies. To the best of the authors’ knowledge, the present study is a pioneer attempt at a time-course urodynamic analysis of TBI rats. In common rodent models, the tonic of the EUS reflects the shutting down of the urethral outlet during the urine storage phase, while burst activity reveals the periodic opening and closing of the outlet to generate a pulsatile flow of urine [16]. In our previous publication, initial CMG results after a TBI revealed very interesting interpretations, and they were divided into two sections of EUS-EMG and CMG. A decreased level of EUS-EMG activity was observed during bladder contractions of an early tonic discharge tailed by a burst pattern (BP) of activity. The burst pattern of activity was categorized by bundles of high-frequency spikes (APs) separated by periods of dormancy (SP). At the time when BP voiding occurred, the intravesical pressure revealed small oscillations [17].

In the present study, the BP of TBI animals started to decreased in all time-course observations and exhibited a significant difference with NC animals. After the induction of a TBI, bladder function was immediately reactivated, and the bladder contractility and urethral activity quickly decreased after the TBI, as reflected in the decreased levels of the BP. TBI animals on day one displayed a drastic reduction in the BP compared to NC rats, and animals from the week-1 group also exhibited similar results. However, this dysfunction was assumed to most likely recover over time, and the time-course study results of the week-2 and -4 groups of animals revealed a gradual increase in the BP compared to NC animals. In addition, AP values immediately dropped compared to NC rats, and this was due to intravesical pressure oscillations that occurred during voiding in NC rats but not in TBI rats. The pressure oscillations played a pivotal role in the EUS pumping activity, which assists effective voiding [18].

SP values were also remarkably reduced in TBI rats on day one immediately after the impact compared to NC animals. This might have been due to the TBI impact, which possibly caused transient electroencephalographic suppression and loss of muscle tone over and above suppression of various reflexes and complexly organized behavior. The mechanism of voiding is a complex process that is closely associated with the cortical region of the brain and includes the motor cortex, somatosensory cortex, cingulate cortex, retrosplenial cortex, thalamus, putamen, insula, and septal nucleus [17,19]. Since a TBI impact damages the cortical area of the brain, eventually the function of the bladder will be disorganized. Values of the BP, AP, and SP all gradually improved in due time but were not fully enhanced for attaining better voiding conditions.

In this study, initial cystometric measurements were recorded between NC rats and TBI rats (Table 1), and from the observed results, it is obvious that VT values increased and CA values decreased after the heavy hammer impact. This scenario mainly was due to initial damage to sensory transmission, which might have elicited a motor consequence resulting in a temporary loss of bladder wall compliance. Since the WDM causes heavy damage to the frontoparietal lobe of the brain, focal modifications of cortical activity or diffuse changes in central blood flow and signal conductivity in this and neighboring areas are the foremost reasons for bladder dysfunction [20]. In addition, damage to the suprapontine region of rats will also result in incontinence, since the detrusor contraction is essentially dependent on sphincter relaxation [21]. Hence, the loss of coordination between the sphincter and detrusor muscles resulted in increased values of VT and decreased values of CA.

The current observations clearly showed that CD values decreased in TBI rats compared to NC rats, and this condition mainly was due to detrusor-external sphincter dyssynergia (DESD). DESD is defined as a spontaneous contraction of the external urethral sphincter over an involuntary detrusor contraction, and the PMC plays a vital role in synchronizing external urethral relaxation prior to it. DESD leads to a circumstance of high storage pressure and incomplete bladder emptying [22]. Significantly increased levels of ICI were observed in TBI rats compared to NC rats. As mentioned earlier, the absence of coordination between external urethral relaxation and detrusor contraction leads to intact sphincter tone. Urinary retention was sustained until larger bladder volumes were attained, and after reaching a certain level, bladder pressure and bladder wall stress subdued the barrier to maintain the urinary flow that was produced by the sphincter muscle [23]. In due course, the presence of a large bladder with improper sphincter tone increased the ICI value. In the present study, results indicated that the RV was remarkably and significantly increased in TBI rats compared to that of NC rats. The PMC plays a crucial role in bladder inhibitory actions [8], and when this center is no longer intact, detrusor hyperreflexia occurs. Two PMCs are respectively responsible for bladder contractions and relaxation, which have reciprocal inhibitory effects. In addition, they also have control over the anterior horns of the sphincter and pelvic floor, and subsequently the pons, which regulates coordination between the detrusor and sphincter [24]. With a TBI, this area might have been severely damaged; hence, urine might not be properly evacuated, which would be a reason for the increased amount of RV. Furthermore, a similar condition was observed in spinal cord injury patients, and the reason was postulated as an increased RV due to upper urinary tract dilation and renal insufficiency [23].

The observed results confirmed that the VV and VE were drastically and significantly reduced in TBI animals compared to NC animals. This situation might have been due to electroencephalographic suppression and loss of muscle tone along with suppression of many reflexes and certain complexly organized behaviors [9]. Since voiding is a complex process and is closely associated with the motor cortex and other cortical regions, it is obvious that the heavy hammer impact will definitely damage this sensitive area, which might directly affect bladder function and abridge the VV and VE in rats immediately after a TBI. Animals belonging to all groups (day 1, week 1, and week 2) exhibited significantly different VEs compared to NC rats; in particular, the week-4 group of animals exhibited a stable condition with remarkably better VE than the other TBI animals, and this indicated that the system could self-recover over time. In addition, as we mentioned earlier, the stability of VE can be further substantiated by the observed results that the difference in VE between the week-2 and -4 groups of animals was very low; thus, the chance for improvement in the VE was very remote. Additionally, a stable urodynamic condition can serve as a suitable model for further TBI-related urodynamic and neuromodulatory studies.

Last of all, the MRI results confirmed the presence of cerebral edema in the cortical region of experimental rats (Figure 2). Cerebral edema can be demarcated as an anomalous accumulation of water molecules within brain tissues and is a major reason for secondary neurological deterioration in subjects with brain injury [25]. It was evident that the TBI induced initial cytotoxic edema followed by ionic and ending with vasogenic edema. The development of cerebral edema is a complex process that includes different phases and may occur in parallel in variously injured brain areas [26]. Cerebral edema eventually leads to intracranial hypertension and is reported to also induce an over-reactive bladder condition [27]. The findings of Salk and Weinstein [28] confirmed that intracranial pressure can decrease the urinary flow rate on the basis of renal vasoconstriction causing a reduction in renal blood flow. Hence, there is a possibility for an over-reactive bladder condition as well as a decreased urinary flow state that can result from cerebral edema. In the current study, the development of cerebral edema was clearly denoted in MRI findings at different phases of the time course. The aforementioned discussion is closely associated with the current findings and also supports them.

Nevertheless, this study has the same limitations as our previous study [23] since the WDM has been standardized in our laboratory, and it was used instead of the lateral fluid percussion impact (LFPI) model. However, the LFPI model rapidly induces severe and transient lower urinary tract dysfunction, and the WDM was chosen because of the favorable results of the MRI showing significant brain damage over the cerebral cortex area in both studies. Furthermore, the current study focused on TBI, which mimics falls or motorcycle road accidents; in this context, none of the other methods exactly mimics similarities as well as the WDM does. Moreover, the present study mainly focused on a time-course analysis of bladder dysfunction in TBI rats; hence, cellular and biochemical parameters were not analyzed. Lastly, the anesthetic effects certainly had a prominent impact on the urodynamic analysis, and it is recognized that urodynamic values will differ between anesthetized rats and freely moving rats. On the other hand, in this study, the results we obtained were highly significant in NC rats as well as TBI rats. Hence, these limitations may have compromised the results, and further studies are warranted to overcome the present limitations.

## 5. Conclusions

In conclusion, the present study divulges the effects of a TBI on the VE at different time intervals. Herein, we elaborated changes in urodynamic parameters that occurred the next day to a 4-week period after an experimental TBI. We also showed that in male rats, the WDM of a TBI led to severe transient or persistent voiding dysfunction. In general, urinary retention, continuous urinary incontinence, and/or alterations in the detrusor volume–pressure relationship are considered to be characteristic features of voiding dysfunction. Most of the above-mentioned distinctive features were observed and are discussed in detail in this study. On the basis of our findings, we concluded that a TBI induces neural dysregulation, which plays a key role in the transmission or translation of afferent signals, and this may be a pivotal reason for bladder dysfunction and a reduced VE. The scenario of the time-course observations clearly showed that the VE drastically decreased after the TBI, and it slowly recovered in due time. But, animals did not regain their VE to the level of NC rats because of the progression of secondary injury. Hence, the progression of secondary injury must be treated or neuromodulatory measures must be engaged to recoup the VE.

## Figures and Tables

**Table 1:** Time-course cystometric measurements between normal control (NC) rats and rats with an experimentally induced traumatic brain injury (TBI).

	VT (mL)	CA (cm H_2_O)	CD (s)	ICI (s)	RV (mL)	VV (mL)	VE (%)
**NC (*n* = 6)**	0.37 ± 0.02	35.21 ± 2.36	20.64 ± 2.82	68 ± 17	0.18 ± 0.04	0.25 ± 0.02	66.4 ± 7
**TBI D 1 (*n* = 7)**	0.77 ± 0.18 *^ψγ^	26.17 ± 4.10 *	16.23 ± 3.22 *	108 ± 20 *	0.59 ± 0.07 *	0.10 ± 0.02 *	14.4 ± 5 *
**TBI W 1 (*n* = 9)**	0.73 ± 0.13 *^ψγ^	24.79± 5.33 *^#^	15.93 ± 2.62 *^#^	108 ± 18 *	0.51 ± 0.05 *^#^	0.12 ± 0.02 *^#^	16.8 ± 5 *^#^
**TBI W 2 (*n* = 9**)	0.66 ± 0.06 *	27.67 ± 3.56 *^ψ^	14.85 ± 3.06 *^ψ^	101 ± 36 *	0.46 ± 0.09 *^#^**^ψ^**	0.20 ± 0.05 *^#^**^ψ^**	31.2 ± 8 *^#^**^ψ^**
**TBI W 4 (*n* = 9)**	0.67 ± 0.08 *^#^	28.00 ± 5.55 *^ψ^	15.78 ± 3.02 *^φ^	96 ± 25 *^#^**^ψ^**	0.28 ± 0.03 *^#^**^ψφ^**	0.22 ± 0.05 *^#^**^ψ^**	32.6 ± 6 *^#^**^ψ^**

^“^*^”^ indicates a significant difference compared with the NC group. ^“**#**”^ indicates a significant difference compared to the day-1 group of animals. “**^ψ^**” indicates a significant difference compared to the week-1 group of animals. “**^φ^**” indicates a significant difference compared to the week-2 group of animals. “**^γ”^** indicates a significant difference compared to the week-4 group of animals. Significance levels are marked at *p* < 0.05. VT, volume threshold; CA, contraction amplitude; CD, contraction duration; ICI, inter-contraction interval; RV, residual volume; VV, voiding volume; VE, voiding efficiency (VV/VT); D 1, day 1; W 1, week 1.
